# Anterior Thalamic Inputs Are Required for Subiculum Spatial Coding, with Associated Consequences for Hippocampal Spatial Memory

**DOI:** 10.1523/JNEUROSCI.2868-20.2021

**Published:** 2021-07-28

**Authors:** Bethany E. Frost, Sean K. Martin, Matheus Cafalchio, Md Nurul Islam, John P. Aggleton, Shane M. O'Mara

**Affiliations:** ^1^School of Psychology and Institute of Neuroscience, Trinity College Dublin, Dublin, D02 PN40, Ireland; ^2^School of Psychology, Cardiff University, Cardiff, CF10 3AS, United Kingdom

**Keywords:** amnesia, diencephalon, hippocampus, memory, space, subiculum

## Abstract

Just as hippocampal lesions are principally responsible for “temporal lobe” amnesia, lesions affecting the anterior thalamic nuclei seem principally responsible for a similar loss of memory, “diencephalic” amnesia. Compared with the former, the causes of diencephalic amnesia have remained elusive. A potential clue comes from how the two sites are interconnected, as within the hippocampal formation, only the subiculum has direct, reciprocal connections with the anterior thalamic nuclei. We found that both permanent and reversible anterior thalamic nuclei lesions in male rats cause a cessation of subicular spatial signaling, reduce spatial memory performance to chance, but leave hippocampal CA1 place cells largely unaffected. We suggest that a core element of diencephalic amnesia stems from the information loss in hippocampal output regions following anterior thalamic pathology.

**SIGNIFICANCE STATEMENT** At present, we know little about interactions between temporal lobe and diencephalic memory systems. Here, we focused on the subiculum, as the sole hippocampal formation region directly interconnected with the anterior thalamic nuclei. We combined reversible and permanent lesions of the anterior thalamic nuclei, electrophysiological recordings of the subiculum, and behavioral analyses. Our results were striking and clear: following permanent thalamic lesions, the diverse spatial signals normally found in the subiculum (including place cells, grid cells, and head-direction cells) all disappeared. Anterior thalamic lesions had no discernible impact on hippocampal CA1 place fields. Thus, spatial firing activity within the subiculum requires anterior thalamic function, as does successful spatial memory performance. Our findings provide a key missing part of the much bigger puzzle concerning why anterior thalamic damage is so catastrophic for spatial memory in rodents and episodic memory in humans.

## Introduction

Bilateral hippocampal formation lesions are the principal cause of the severe and enduring “temporal lobe amnesic” syndrome (Spiers et al., 2001; Aggleton and Morris, 2018). Bilateral medial diencephalic lesions can also cause a severe and enduring memory syndrome (“diencephalic amnesia”), which can closely resemble temporal lobe amnesia (Kopelman et al., 1995; Aggleton, 2008). Studies of Korsakoff's syndrome and analyses of thalamic strokes show the anterior thalamic nuclei (ATN: the anteromedial, anteroventral, and anterodorsal nuclei) appear most critical for diencephalic amnesia (Harding et al., 2000; Van der Werf et al., 2003; Carlesimo et al., 2011; Segobin et al., 2019). Mammillary body damage may also contribute to diencephalic amnesia (Dusoir et al., 1990; Tsivilis et al., 2008), with mnemonic actions via the ATN (Aggleton and Brown, 1999; Vann, 2010).

Theories of temporal lobe amnesia assume that the hippocampal formation is vital for the brain's cognitive mapping capabilities (Colgin, 2020) and is the core of an extended system vital for human episodic memory (Spiers et al., 2001; O'Mara and Aggleton, 2019). This network model suggests that the ATN are key structures within both diencephalic and temporal lobe memory systems (Aggleton and Brown, 1999; Ranganath and Ritchey, 2012). In rodents, for instance, anterior thalamic lesions severely impair spatial learning, mirroring hippocampal damage (Morris et al., 1982; Warburton and Aggleton, 1999; Moran and Dalrymple-Alford, 2003); moreover, disconnection studies suggest that the hippocampal formation and ATN are functionally interdependent (Sutherland and Rodriguez, 1989; Shibata, 1993; Nelson et al., 2020).

The hippocampal formation and ATN have direct, reciprocal connections via the subiculum (Shibata, 1993), prompting whether anterior thalamic pathology disrupts hippocampal formation functioning, thereby impairing learning and memory. The subiculum also receives indirect ATN inputs via the entorhinal cortices, presubiculum, and retrosplenial cortex. The strategic importance of the subiculum is further evident as it distributes many hippocampal projections beyond the temporal lobe (Meibach and Siegel, 1977; Jay and Witter, 1991; Aggleton, 2012).

Although adjacent, subiculum and hippocampal area CA1 have differing properties. For example, CA1 contains numerous place cells, whereas the subiculum contains a lower frequency, but greater variety, of spatial cells (place, grid, head-direction, and boundary vector cells) (Thompson and Best, 1989; Brotons-Mas et al., 2010, 2017; Meshulam et al., 2017; Broussard et al., 2020). Furthermore, the ATN (especially the anterodorsal nucleus) are a vital source of head-direction information (Taube, 1995; Tsanov et al., 2011); anterodorsal thalamic nucleus lesions eliminate parahippocampal head-direction signals and disrupt parahippocampal grid cell activity (Winter et al., 2015; Peyrache et al., 2019). Nevertheless, head-direction network disruption does not explain the amnesia associated with anterior thalamic lesions because lesions of the head-direction system cause only mild, often transient, qualitatively different, spatial deficits compared with the severe, permanent deficits seen after lesions of ATN (Aggleton et al., 1996; Byatt and Dalrymple-Alford, 1996; Dillingham and Vann, 2019).

Given the close anatomic relationship between the subiculum and the ATN, we examined how permanent and reversible ATN lesions might affect subicular spatial coding and spatial alternation memory. Moreover, hippocampal area CA1 projections to the subiculum show LTP (O'Mara et al., 2000). We therefore made additional recording comparisons with hippocampal area CA1 which, like the subiculum, projects to cortical sites beyond the temporal lobe, but does not receive direct afferents from the ATN.

We found that permanent and transient rat anterior thalamic lesions (sparing nucleus reuniens) abrogate spatial signaling by the subiculum. Paradoxically, place coding by CA1 cells remained intact after anterior thalamic lesions, despite spatial alternation dropping to chance levels. The observed silencing seemingly applies to all subicular spatial cell types (place, grid, border, head-direction). Further, because the subiculum is a key source of hippocampal projections beyond the temporal lobe (Meibach and Siegel, 1977; Irle and Markowitsch, 1982; Jay and Witter, 1991), the impact of anterior thalamic damage as a key component of diencephalic amnesia is amplified.

## Materials and Methods

### 

#### Key resources

Key resources are listed in Table 1.

**Table 1.** Key resources

Reagent or resource	Source	Identifier
Antibodies		
Mouse monoclonal anti-Calbindin	Swant	Catalog #300
Mouse monoclonal anti-NeuN (clone A60)	EMD Millipore	Catalog #MAB277, Lot #2829834
Mouse monoclonal anti-Parvalbumin	Swant	Catalog #PV235
Rabbit polyclonal anti-Fluorescent Gold	EMD Millipore	Catalog #AB153-1, Lot #2905401
Biotinylated horse anti-rabbit IgG (H + L)	Vector Laboratories	Catalog #BA-1100
Biotinylated horse anti-mouse IgG (H + L)	Vector Laboratories	Catalog #BP-2000
Critical commercial assays		
DAB peroxidase (HRP) substrate kit (with nickel), 3,3′-diaminobenzidine	Vector Laboratories	Catalog #SK4100
Vectastain Elite ABC HRP Kit (peroxidase, Standard)	Vector Laboratories	Catalog #PK6100
Deposited data		
Raw and analyzed data	This paper	https://osf.io/vdakx/
Experimental models: organisms/strains		
Rat: Lister hooded (male)	HsdOla:LH	Envigo
Software and algorithms		
FIJI (Fiji Is Just ImageJ)	Schneider et al., 2012	https://imagej.net/Fiji
TINT	Axona	http://axona.com/
R Statistics	R Core Team, 2017	https://www.r-project.org/
NeuroChaT	Islam et al., 2019	https://github.com/shanemomara/omaraneurolab
Other		
25 µm platinum-iridium wire	California Fine Wire	Catalog #100167

#### Subject details

Experiments were conducted on 23 male Lister Hooded rats (Envigo) with preprocedural weights of 309-356 g. Upon arrival, animals were cohoused on a 12 h day/night cycle and handled daily by the experimenter for a week before surgical procedure. Before surgery and during recovery, animals had free access to food and water; during behavioral testing, food was restricted but ensured the animals did not fall below 85% of the animal's free feeding weight. All rats were naive before the present study. Selection of animals between lesion and control groups was alternated according to body weight before surgery (starting with the heaviest), so that preprocedural weights were matched, and the groups balanced.

#### Experimental design

During stereotaxic surgery, rats were implanted unilaterally with 28 electrodes of 25 µm thickness platinum-iridium wires (California Fine Wire) arranged in a tetrode formation. Tetrodes were targeted at the dorsal subiculum, CA1, or at the subiculum and CA1 simultaneously. An additional bipolar electrode (stainless steel, 70 µm thickness) targeting the ipsilateral retrosplenial cortex was also implanted in all but 2 cases (see below), but the data from these electrodes are considered elsewhere. All electrodes were connected to a 32-channel microdrive (Axona).

For the permanent lesion experiments, 7 animals received stereotaxic cytotoxic lesions targeting the ATN (ATNx), followed by electrode implantation targeting dorsal subiculum and retrosplenial cortex. Meanwhile, 3 rats underwent sham injections of equivalent volumes of PBS only (Sham controls). A further 4 rats (Normal controls) had no sham lesion procedure (i.e., just had electrodes implanted). Both the sham and normal controls had electrodes targeting dorsal subiculum.

The temporary inactivation (muscimol) experiment followed the permanent lesion study. For this, a further 6 animals (ATNmusc) were implanted with tetrode and bipolar electrode configurations alongside bilateral infusion cannulae (26 gauge, 4 mm length, Bilaney Consultants) in the ATN. Electrodes were positioned as above, with two exceptions; 1 animal was implanted with four tetrodes targeting the subiculum, three targeting CA1 and one targeting retrosplenial cortex, with no additional bipolar electrode; and 1 rat was implanted with tetrodes targeting the subiculum and the bipolar electrode targeting CA1.

Finally, for the CA1 experiment, a further 3 rats (ATNx_CA1) received permanent bilateral cytotoxic ATN lesions followed by electrode implantation into CA1.

##### Ethics

All experimental procedures were in accordance with the ethical, welfare, legal, and other requirements of the Healthy Products Regulatory Authority regulations and were compliant with the Health Products Regulatory Authority (Irish Medicines Board Acts, 1995 and 2006) and European Union directives on Animal Experimentation (86/609/EEC and Part 8 of the EU Regulations 2012, SI 543). All experimental procedures were approved by the Comparative Medicine/Bioresources Ethics Committee, Trinity College Dublin, Ireland before conduct, and were conducted in accordance with LAST Ireland and international guidelines of good practice.

##### Surgical methods: permanent ATN lesions and electrode placements

Rats were first anesthetized with isoflurane (5% to induce anesthesia, 1%-2% to maintain) combined with oxygen (2 L/minute). After being placed in a stereotaxic frame, chloramphenicol 0.5% eye gel, preoperative antibiotics (Enrocare, 0.1 ml in 0.5 ml saline) and analgesia (Metacam, 0.1 ml) were administered.

The skull was exposed, and connective tissue removed. For the ATNx cohort (*n* = 7), bilateral neurotoxic lesions targeting the ATN were performed using slow infusions of 0.12 m NMDA dissolved in PB solution (PBS, pH 7.35). NMDA was infused over 5 min (0.22 or 0.24 µl per site) via a 0.5 µl Hamilton syringe (25 gauge), with the syringe left in position a further 5 min at each of four target sites before slow retraction. The craniotomies were then sealed using bone wax (SMI). The ATN lesion coordinates, with the skull flat, were as follows from bregma: AP −1.7 mm, ML ±0.8 mm, DV −5.7 mm from top of cortex; AP −1.7 mm, ML ±1.6 mm, DV −4.9 mm from top of cortex. Sham control animals (*n* = 3 rats) underwent four equivalent infusions of PBS only.

Bundles of 28 electrodes of 25 µm thickness platinum-iridium wires (California Fine Wire) arranged in a tetrode formation were implanted unilaterally. Tetrodes were implanted aimed at the dorsal subiculum (AP −5.6 mm, ML 2.5 mm, DV −2.7 mm from top of cortex), CA1 (AP −3.8 mm, ML 2.5 mm, DV −1.40 mm from top of cortex), or both. Electrodes were stabilized with dental cement (Simplex Rapid) attached to the screws implanted into the skull. An additional bipolar electrode (stainless steel, 70 µm thickness) targeting the ipsilateral retrosplenial cortex was also implanted in all but 2 cases, the data from which are considered elsewhere. All electrodes were connected to a 32-channel microdrive (Axona).

For the ATN inactivation experiment, a further 6 animals (ATNmusc) were implanted with tetrode and bipolar electrode configurations alongside bilateral infusion cannulae (26 gauge, 4 mm length, Bilaney Consultants). Cannulae were placed targeting ATN (AP −1.7 mm, ML ±3.8 mm, DV −4.0 mm from top of cortex, at angle 28.6° toward center), then fixed in position using dental cement and dummy cannulae inserted to prevent blockage. Electrodes were positioned as above, targeting dorsal subiculum and RSC, with two exceptions; 1 animal was implanted with four tetrodes targeting the subiculum, three targeting CA1 and one targeting retrosplenial cortex, with no additional bipolar electrode; and 1 rat was implanted with tetrodes targeting the subiculum and the bipolar electrode targeting CA1.

Glucosaline (5-10 ml) was administered subcutaneously postoperatively and the animal allowed to recover. Animal weight, activity, and hydration were closely monitored daily for a minimum of 7 d before beginning electrophysiological recordings.

##### Electrophysiological recordings

Electrophysiological recordings were obtained using an Axona Ltd 64-channel system, allowing dual recordings of single units and local field potentials from each electrode. Initial habituation recordings were conducted in a 60 × 60 cm square, walled arena (height 42 cm). Later testing involved a larger arena (105 × 105 cm, 25 cm height). For both arenas, the walls and floors were made of wood painted matt black. A black curtain could be closed around the arena to remove distal spatial cues, and visual cues could be attached to the curtain as required. The habituation sessions in the small arena allowed the animal to acclimatize to the recording procedure and the experimenter to adjust electrode locations until the optimal recording depth was reached. After the habituation period (usually 3–7 d), rats were first trained on the behavioral tasks (T-maze then bow-tie maze) with up to 1 h of free exploration with pellet-chasing tasks typically recorded afterward in the same day. Pellet-chasing included the rotation of spatial cues on the curtain during the recording of single-unit activity in the small arena to examine whether spatial units remap accordingly, exploration in the small, then large, arena to assess spatial cell remapping, and consecutive recordings to examine sleep properties (data not described here).

#### Behavioral tasks

##### Spatial alternation

A four-arm cross-shaped wooden maze with raised sides (119 × 119 cm full length; each arm 48 × 23 cm; height 30 cm) was used for the spatial alternation task, allowing the rotation of start points. Each arm could be blocked close to the center to form a T-maze. In addition, a barrier could be placed within an arm to form a holding area for the start position. Distal spatial cues were available in the recording room, including the pulled-back curtain, electrophysiological recording equipment set on wall-mounted shelves, a desk and computer. Animals were first habituated in pairs to the maze and allowed to freely explore for 10 min. Rats then individually had two pretraining sessions (5 min each) in which they were first placed behind a barrier at the start position at one end of the maze, retained for 10 s, then the door removed and the rat allowed to explore the maze. The maze arm opposite to the start was blocked, and sucrose pellets (TestDiet 5TUL 20 mg) were placed in a shallow dish at the end of each open arm so that they were not visible from the center of the maze. Rats learned to run to an arm of the maze to obtain sucrose pellets, which were replaced once they had been consumed and the animal left that arm. The maze was then altered so that a different start arm and blocked arm were used, and another training session run. Each rat had four 5 min training sessions per day such that each start/blocked configuration was experienced (opposite, adjacent, opposite) for 2 d initially, with an additional day if required.

For the experiment, rats were placed in the start position for 10 s. In each trial, there was an initial forced run (“sample”), in which two arms of the cross-shaped maze were blocked, forcing the animal into either the left or right arm to obtain two sucrose pellets from the end of the arm. The animal was then picked up and returned to the start position and held for ∼20 s before being released for the choice run (“test”), in which one of the barriers was removed from the maze so that the animal had the choice of either the left or right arm (see Fig. 2*A*). Sucrose pellets were only available in the arm opposite to the sample run, so the rat was rewarded if it alternated. A choice was determined when the back paws of a rat had entered the arm. The rat was then removed from the maze and placed in an open holding cage for 2-3 min while the maze was rearranged. Each animal had eight sessions consisting of eight trials each, with both the start position and forced turn pseudorandomized so that the same arrangement did not occur more than twice consecutively. Rats were connected to the electrophysiological recording equipment throughout pretraining and each test session.

##### Bow-tie maze

The bow-tie maze allows continuous object recognition testing, with multiple trials and new novel objects during each session (Albasser et al., 2010). The bow-tie-shaped maze had raised sides (wood painted matt black; 120 cm long × 50 cm wide, 50 cm height) and a central sliding door. Partitions at each end of the maze split both ends into two short corridors (see Fig. 2*C*). The animals were first habituated to the maze for 10 min, by allowing free exploration with sucrose pellets scattered throughout. Next, during the 10 min session, pellets were first placed in wells at the two ends of the maze and the rat trained to run from one end of the maze to the other when the central door was opened. Then, opaque plastic objects (a funnel and a beaker) were placed behind each baited well. The objects were gradually moved so that they increasingly covering the wells. Rats underwent 4 or 5 10 min pretraining sessions, until they readily shuttled across the maze to retrieve sucrose pellets by pushing objects.

For the experimental procedure, 22 pairs of novel objects were used. In the first trial, one novel object was placed covering the sucrose pellets. The rat retrieved the pellets and investigated the object at that end of the maze. After 1 min, the central door was opened and the rat passed to the opposite end of the maze, where there would be a repeat of the original object (now familiar) and a new novel object (both covering sucrose pellets). This procedure was repeated for all 22 pairs of objects, so that each of the 21 trials consisted of a new novel object and the previous object, which was now familiar (Albasser et al., 2010). Animals were video recorded throughout.

For analysis, the time spent investigating each object was recorded and two measures of recognition, D1 and D2, were calculated (Albasser et al., 2010). D1 represents the difference in exploration time between novel and familiar objects, and is calculated by subtracting the time spent exploring a familiar object from the time spent exploring a novel object. Cumulative D1 represents the sum of the exploration time for all novel objects minus the sum of exploration time for all familiar objects across all trials. D2 represents the total difference in exploration time (cumulative D1) divided by the total exploration time for both novel and familiar objects, resulting in a ratio that ranges between −1 and 1.

#### Transient inactivation of the ATN (muscimol)

An additional cohort (*n* = 6, ATNmusc) was implanted with bilateral infusion guide cannulae (26 gauge, 4 mm length; Bilaney Consultants) aimed at the ATN at an angle of 28.6° toward the midline (AP −1.7 mm, ML ±3.6 mm, DV −4.0 mm from top of cortex) alongside subiculum electrode implantation. A dummy cannula (0.203 mm diameter, 4 mm length; Bilaney Consultants) was used to protect each guide cannula during recovery and normal recording activity. All other surgical and electrophysiological methods were the same as those used for the animals with permanent ATN lesions.

Following recovery, rats were trained daily for a minimum of 1 week before commencing inactivation experiments. Rats were lightly restrained and the dummy cannulae removed and replaced several times. During this period, rats were also trained in the spatial alternation (T-maze) task and electrophysiological activity was recorded during free exploration and pellet-chasing in the large and small square arenas. All apparatus matched that used for the ATNx rats.

**Figure 1.**
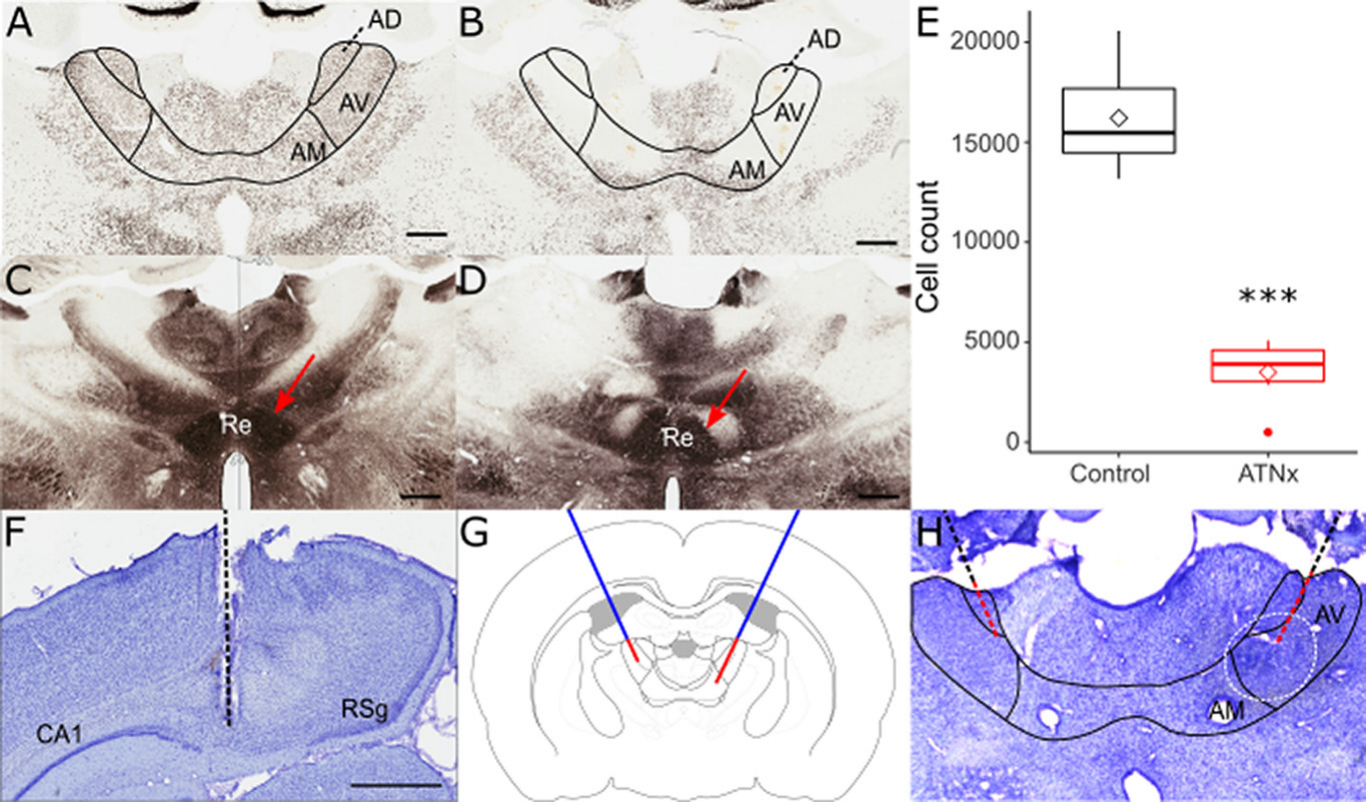
NeuN-reacted coronal sections showing the status of the AV (anteroventral), AM (anteromedial) and AD (anterodorsal) thalamic nuclei in control (***A***) and lesion (***B***) animals. The nucleus reuniens (Re, arrowed), as shown using calbindin-reacted sections, was intact in both control (***C***) and lesion (***D***) animals, indicating that reuniens damage was not responsible for deficits seen in ATN-lesioned animals. ***E***, The ATN cell count was significantly reduced in lesioned animals (ATNx) compared with controls (Control). Nissl-stained coronal sections helped to confirm electrode placement in dorsal subiculum (***F***), with the electrode path indicated. ***G***, Schematic representing cannula placement (blue) and the two targets of the infusion needle (red). ***H***, Cresyl violet-stained section indicating cannula placement, with DAB-reacted Flurogold infused to indicate spread of muscimol. Black line indicates canula placement. Red line indicates the track of the infusion needle. Dashed white line indicates the spread of the muscimol. ***p < 0.001 (Welch's two-sample t test). Scale bar, 800 mm. RSg, retrosplenial cortex.

On the day of experimentation, electrophysiological recordings of exploration and pellet-chasing were first performed before infusion for 20–40 min to establish a baseline. The animal was then lightly restrained and muscimol (concentration 0.5 mg/1 ml saline) infused through a 33 gauge infusion needle with a 1 or 2 mm projection past the length of the implanted guide cannula, targeting the ATN. Muscimol was infused over 90 s using a 0.5 µl Hamilton syringe and infusion pump (KD Scientific). The infusion needle was retained in position for a further 60 s before it was removed and replaced with the dummy cannula. Rats received a 0.2 µl at each of two locations per hemisphere using both a 5 and 6 mm length infusion needle to target the whole ATN (see Fig. 1*H*). Following infusion, electrophysiological recordings during pellet-chasing and exploration were conducted in consecutive 20 min sessions in the small and large square arena. Arenas were swapped between recordings to maintain the animal's interest in the surroundings and to assess whether spatial remapping occurred. Between 90 and 120 min into the experiment, the T-maze task was performed (8 trials, pseudo-randomized starting points). Animals were then returned to the square arena and recorded for a further 2-3 h, including during sleep. Regular diet and water were freely available in the recording arena after the T maze test. The following day, further electrophysiological recordings were undertaken to determine whether the effects of muscimol had ceased and cell activity had returned to baseline.

Muscimol infusions were repeated 1-2 weeks after the initial experiment. In 2 cases, an additional control infusion of saline was given. To visualize the location of the muscimol infusion, the tracer Flurogold (Sigma-Aldrich) was infused 1 d before perfusion.

#### Perfusion and histology

After completion of experiments, animals were killed and perfused transcardially with 0.1 m PBS and then 2.5% PFA in 0.1 m PBS. Brains were removed and postfixed in PFA for 24 h and then transferred to 30% sucrose in 0.1 m PBS solution for 2 d. A cryostat (Leica Microsystems CM1850) was used to cut 40 µm sections in a 1:4 series. One series was mounted onto double gelatin-subbed microscope slides and, once completely dry, washed in decreasing concentration of alcohol (100%, 90%, 70%) before being stained with cresyl violet, a Nissl stain (Sigma-Aldrich). Sections were then dehydrated with increasing alcohol concentrations, washed in xylene, and coverslipped.

Of the other series, one was reacted against anti-calbindin antibody raised in mouse (Swant) and another against anti-NeuN antibody raised in mouse (EMD Millipore). The remaining series was reacted with either anti-parvalbumin antibody raised in mouse (Swant) or, in the temporary inactivation cohort, with anti-Flurogold raised in rabbit (EMD Millipore).

In brief, sections were washed in a quench solution (10% methanol and 0.3% hydrogen peroxide in distilled water) before 0.1 m PBS, pH 7.35, then PBST (2 ml Triton X-100 in 1 L 0.1 m PBS; pH 7.35) washes. Sections were stirred for 1 h in 4% normal horse serum in PBST before the primary antibody was added (1:5000 dilution in PBST for calbindin, parvalbumin, and Flurogold; 1:10,000 for NeuN) and stirred at 4° overnight. Sections were then washed in PBST before being incubated for 2 h in 1:250 dilution of horse-anti-mouse (Vector Labs) or, in the case for Flurogold, horse-anti-rabbit (Vector), in PBST. After further PBST washes, sections were incubated at room temperature in Vectastain Elite ABC Solution (Vector Labs) before further PBST and PBS washes. Sections were then reacted with DAB solution (Vector Labs) and washed in PBS.

All sections were mounted on double-subbed slides, and Flurogold-reacted slides were lightly stained with cresyl violet for improved tissue visualization before coverslipping. Sections were imaged using either an Olympus BX51 upright microscope or Leica Microsystems Aperio AT2 slidescanner.

#### Statistical analyses

##### Behavioral analysis

To analyze T-maze results, the mean score was compared between Normal Control, Sham Control, and ATNx groups using ANOVA with Tukey *post hoc* test. For the muscimol experiments, T-maze results during ATN inactivation were compared with the same animals before inactivation.

To analyze object recognition using the bow-tie maze, the time spent investigating each object was recorded and two measures of recognition (D1 and D2) were calculated (Albasser et al., 2010).

##### Unit identification and isolation

Spike sorting was performed automatically in Tint using *k*-means (Axona) and cluster cutting refined manually. Unit identification used the following criteria: units had to be active and show consistent waveform characteristics (amplitude, height, and duration) during recording, as well as a clean refractory period (>2 ms) in the interspike interval (ISI) histogram. Spike amplitude was measured as the difference between the positive peak and the first negative peak before the positive peak, if present, or zero. Spike height was the difference between the spike peak to the minimum value of the spike waveform. Spike width was the distance in microseconds beyond which the waveform drops below 25% of its peak value. Histograms were used to assess spike width and determine whether qualitatively different neuron populations were being recorded following anterior thalamic lesions.

Units were sometimes seemingly recorded for >1 d, despite electrode lowering. For these cases, cells were monitored on the relevant tetrodes from day to day; for analysis, only clean recordings with the largest sample size and spikes of the highest amplitude were chosen. To avoid double-counting cells, care was taken to exclude seemingly related samples from analysis. During spike sorting, the signals from each cell were carefully followed from first appearance to complete loss, to avoid overestimation of cell counts. Once well-defined neuronal signals were isolated, recording commenced. For permanent lesion experiments, rats had to explore at least 90% of the arena in a session to be included in analyses to allow reliable calculation of spatial characteristics.

Standard statistical testing was performed using an open-source custom-written suite in Python (NeuroChaT; available for download at https://github.com/shanemomara/omaraneurolab, https://github.com/shanemomara/NeuroChaT) (Islam et al., 2019), and additional custom codes in R (R Foundation for Statistical computing; https://www.r-project.org). Units were classified based on the spatiotemporal features of their activity in the arena during pellet-chasing, as described below.

##### Bayesian analysis

To investigate whether the apparent absence of spatial signal in ATNx animals was significant, a Bayesian approach was applied as follows. Let PC denote the probability that a control recording will contain a spatial cell, and PL denote the probability that a lesion recording will contain a spatial cell. Let D denote the recorded data. By Bayes theorem, P(PC, PL | D) = P(D | PC, PL) *P(PC, PL)/P(D) (i.e., posterior = likelihood *prior/probability of the evidence). We use a uniform prior distribution, as we have no prior belief of how common recordings in the subiculum with spatial cells are, so P(PC, PL) = 1. The probability of the evidence is a constant normalizing parameter, which can be calculated by ensuring the posterior distribution sums to 1. By the independence assumption of selected recordings, the likelihood function can be modeled as the product of evaluating the probability mass functions arising from two binomial distributions as follows:

$$P\text{(}D\text{ | }PC,PL)=\left(\begin{array}{c} CR\\ CS \end{array}\right)PC^{CS}{\left(1-PC\right)}^{CR-CS}\left(\begin{array}{c} LR\\ LS \end{array}\right)PL^{LS}{\left(1-PL\right)}^{LR-LS}$$

Where the data D provide the information, CR the number of control recordings, CS the number of control recordings with a spatial cell, LR the number of lesion recordings, and LS the number of control recordings with a spatial cell. As an example, using the data from the contingency table for the recordings with spatial cells (Table 2) as follows:

$$P\text{(}D\text{ | }PC,PL)=\left(\begin{array}{c} 53\\ 15 \end{array}\right)PC^{15}{\left(1-PC\right)}^{53-15}\left(\begin{array}{c} 47\\ 0 \end{array}\right)PL^{0}{\left(1-PL\right)}^{47-0}$$

**Table 2.** Recording counts from control and ATNx animals^*a*^

Observed frequencies for recordings	Control	Lesion	Total
Spatial cells			
At least one spatial cell was recorded	15	0	15
Spatial cells were not recorded	38	47	85
Total recordings performed	53	47	100
Nonspatial cells			
At least one nonspatial cell was recorded	16	13	29
Nonspatial cells were not recorded	37	34	61
Total recordings performed	53	47	100

^*a*^Recordings from Control and ATNx animals (where a “recording” is defined as a single trial of an open field recording session) were selected such that recordings were independent. Recordings were deemed to be independent if they were performed before and after adjustment of electrode position, or if they were several days apart, as the electrodes had likely shifted naturally. For each of these recordings, the presence or absence of both spatial cells and nonspatial cells was marked, and the observed frequencies are presented.

##### Burst properties

Bursting units were identified using criteria based on Anderson and O'Mara (2003). A burst was defined as a series of spikes in which each ISI was a minimum of 6 ms, and contained a minimum of two spikes, with a minimum interburst interval (IBI) of 50 ms. Further bursting analyses examined the total number of bursts during a recording session, the number of spikes in the bursting cluster, mean ISI during the burst cluster, number of spikes per burst, burst duration, duty cycle (the portion of an IBI during which a burst fires), the IBI, and propensity to burst, calculated by dividing the number of bursting spikes by the total number of spikes in a recording.

##### Spatial analyses

Additional analyses examined spatial modulation of recorded units. Multiple indices were used to analyze the spatial properties of unit activity (i.e., spatial coherence, spatial information content, and spatial sparsity). A firing field was defined as a set of at least nine contiguous pixels with firing rate >0. A place field was identified if nine neighboring pixels (sharing a side) were >20% of the peak firing rate. Place field size was represented by number of pixels. Spatial specificity (spatial information content) was expressed in bits per spike (Skaggs et al., 1996). Mean spiking frequency is the total number of spikes divided by the total recording time and is expressed in spikes/s. Exploration was assessed by comparing the occupancy of bins and the number of visits per bin during recording sessions. Additionally, to be regarded as place cells, the following criteria had to be met: all included as place cells had to have a spatial information content (Skaggs et al., 1996) index of >0.5, a spatial coherence of >0.25, and a mean firing rate of >0.25. The spatial path of the subject and the spike train were used to produce a locational firing rate map.

To analyze head direction cells, the animal's head direction was calculated using the relative position of two tracked LEDs on a bar attached to the microdrive, in the horizontal plane. The directional tuning function was determined by plotting the firing rate as a function of the head direction divided into 5° bins, and the firing rate calculated by the total number of spikes divided by time spent in each bin.

To determine the existence of a hexagonal grid firing structure, grid index, size, and orientation were calculated. The grid cell analysis included calculating the spatial autocorrelation of the firing rate map and assessing the shape formed by the peaks in autocorrelation. For border cell analyses, cells with a firing profile that was parallel to the border of the arena were selected by plotting the positional firing pattern.

##### Downsampling analysis

We performed a spatial downsampling procedure as a control method to rule out the possibility that the disruption in spatial firing merely reflects a lack of sampling of the environment, given that the animals move less distance and cover less of the environment while under the effects of muscimol.

Our method is based on the spatial downsampling performed by Boccara et al. (2019): for each cell, we produce a list *L = (x_t, y_t, spike_t)* where *x_t, y_t* is the position of the animal at time *t* and spike_t is the number of spikes the cell emitted in that time bin. The procedure to spatially downsample data from recording A to match the exploration in recording B is as follows. First, the list L from B is binned into 3 cm squares based on x_t, y_t and the list L from A is binned using the same number of bins as was used for B. Second, several random samples are drawn (with repetition) from the list L in A such that the number of samples drawn from an individual bin is the minimum of the number of data points falling in that bin between recordings A and B. Using this spatially downsampled data, firing rate maps and spatial statistics are performed as in the rest of this paper. This procedure is performed 200 times for each cell.

Using the method above, recordings were spatially downsampled to match their own spatial occupancy as an additional control since the random sampling procedure should cause small changes. After testing spatial coherence, spatial information content, and spatial sparsity, coherence was the only measure tested that was resistant to downsampling against self (mean decrease of 0.015) and downsampling to other random data (mean decrease of 0.05).

For each cell considered in the muscimol experiments, the baseline recording was downsampled to match the muscimol recordings, and the muscimol recordings were downsampled to match the baseline recording. These resulting firing maps had closely matching occupancy of the environment, and coherence was computed on these maps.

##### Image analysis

Individual images of sections were aligned and tiled using Inkscape (http://inkscape.org) and FIJI (Fiji Is Just Image J, https://imagej.net/Fijii) (Preibisch et al., 2009). Cresyl violet-stained sections helped to confirm electrode placement. To assess lesion success, the ATN was segmented from photomicrographs of anti-NeuN-reacted sections using FIJI and the resulting image thresholded to show nuclei separation, before using the inbuilt Analyze Particles plugin to obtain a cell count. The ATN cell counts were compared between the ATNx and control groups to determine lesion effectiveness. Calbindin-reacted sections helped to determine the status of nucleus reuniens.

#### Statistical analysis

Statistical analyses were performed in R Statistics (https://www.r-project.org/). No statistical difference was noted between Normal Control and Sham Control groups, so these were combined into a single Control group unless otherwise stated. Distribution of variables was assessed using histograms; and where a normal distribution was determined, differences between groups were examined using Welch's two-sample *t* test, an adaptation of Student's *t* test that is more reliable when samples have unequal variances or sample sizes. Where three groups were compared, ANOVA with a Tukey *post hoc* test was applied. In cases where the distribution of a variable was nonparametric, differences between groups were assessed using Mann–Whitney *U* test.

For temporary inactivation experiments, data were compared with baseline (immediately before muscimol infusion) values using Welch's two-sample *t* test with a Bonferroni correction to account for multiple comparisons and reduce the likelihood of Type 1 errors.

Statistical tests are reported in the text and appropriate figure legends. Boxplots represent median, 25th, and 75th percentiles. Boxplot tails represent the smallest and largest value within 1.5 times the interquartile range, and outliers are defined as values that are >1.5 times and <3 times the interquartile range. Data means are represented with a diamond-shaped marker. Error bars on scatter plots indicate SEM.

#### Data and code accessibility

Datasets generated during this study are available at OSF (https://osf.io/vdakx/), and code is available at GitHub (https://github.com/shanemomara/omaraneurolab). Further information will be available on request by contacting S.M.O'M.

## Results

Throughout, the terms hippocampal formation and hippocampal refer to the dentate gyrus, the CA fields, and the subiculum. The parahippocampal region includes the presubiculum, parasubiculum, postsubiculum, and entorhinal cortices. Recordings of neuronal activity were conducted in the subiculum and area CA1. Spatial alternation memory and object recognition memory were assessed using a T-maze and a bow-tie-shaped maze, respectively. Rats received permanent neurotoxic lesions of the ATN (ATNx) following injections of NMDA, or transient ATN lesions following infusion of muscimol. Other rats served as controls.

### Combining ATN lesions (NMDA) with implantations in the dorsal subiculum

Of 23 animals implanted, 1 ATNx rat was subsequently excluded because of electrode malfunction, and 1 Normal Control was excluded because of postsurgical complications. Comparisons between the Normal Control and Sham Control groups consistently failed to reveal group differences and, for this reason, they were typically combined (group “Control”).

### NMDA injections caused considerable cell loss within the ATN, but spared nucleus reuniens

Lesion effectiveness was quantified by comparing the total anti-NeuN-reacted cell counts in 40-µm-thick brain sections containing the anteromedial, anterodorsal, and anteroventral thalamic nuclei in ATNx animals with the corresponding Control values (Fig. 1*A*,*B*). The ATNx rats had markedly reduced cell counts (Control 16,209 ± 2507, ATNx 3497 ± 1528; *t*_(8.26)_ = −9.68, *p* < 0.001, Welch's two-sample *t* test; Fig. 1*E*), while the calbindin-reacted sections helped to confirm that nucleus reuniens remained intact (Fig. 1*C*,*D*), indicating that, despite a significant role in spatial working memory (Griffin, 2015), nucleus reuniens damage was not a contributing factor to differences in either behavior or cell firing properties in ATNx animals.

### ATN lesions (NMDA) reduce spatial alternation memory to chance levels of performance

Unless otherwise stated, Welch's two-sample *t* tests were performed to compare Control and lesion data.

**Figure 2.**
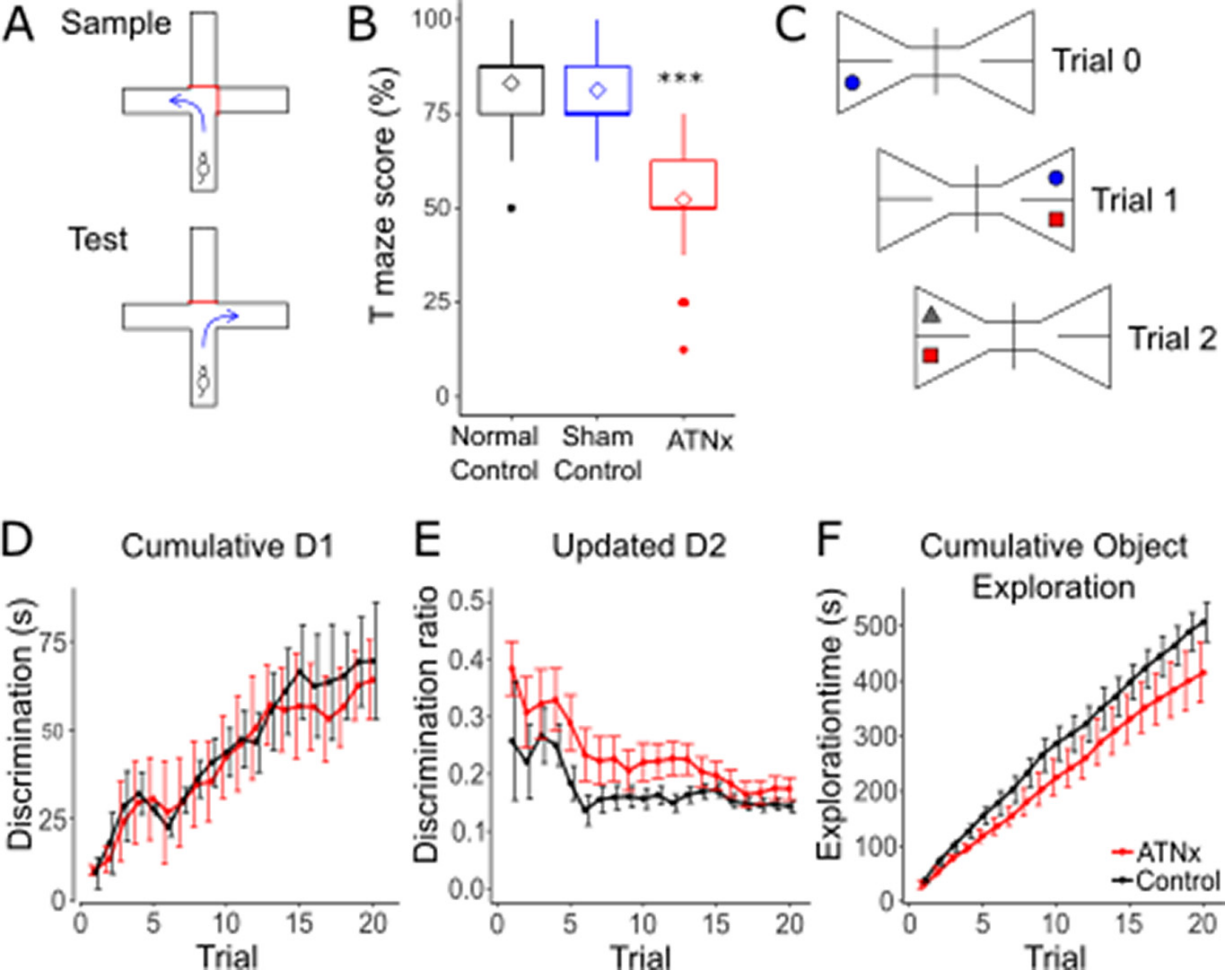
***A***, Schematic diagram of spatial alternation task. ATN-lesioned animals (ATNx) showed a significant deficit in spatial alternation compared with both control and sham animals (***B***). ***C***, Schematic of novel object recognition task. There was no difference between control (Control) and lesion rats in cumulative D1 (***D***), D2 (***E***), or total exploration time (***F***). ***p < 0.001 (ANOVA with Tukey *post hoc*). Error bars indicate SEM.

Consistent with previous lesion studies, ATNx animals had considerably lower spatial alternation scores (52.23 ± 14.58%), compared with Normal (83.10 ± 10.96%) and Sham (81.25 ± 11.41%) controls (ANOVA, *F*_(2,114)_ = 77.8, *p* < 0.001; Tukey *post hoc p* < 0.001; Fig. 2*B*), confirming their severely impaired spatial working memory. In contrast, ATNx animals showed no impairment in novel object discrimination compared with the Control animals (cumulative D1 *t*_(142.69)_ = 0.99, *p* = 0.324; updated D2 *t*_(172.25)_ = 1.42, *p* = 0.159), indicating that recognition memory under these conditions remains intact (Fig. 2*D–F*). During free exploration in the square arenas with electrophysiological recordings, the ATNx rats traveled greater distances than the Control animals both during habituation (Control 162.14 ± 17.43 m, ATNx 85.45 ± 14.21 m; *t*_(16.14)_ = 3.55, *p* = 0.003) and during subsequent recordings (Control 120.66 ± 15.19 m, ATNx 142.41 ± 10.89 m; *t*_(14.55)_ = 2.98, *p* = 0.096), suggesting slightly increased motor activity after ATNx.

### ATN lesions (NMDA) abrogate spatial firing in the subiculum only: quantitative analyses

**Figure 3.**
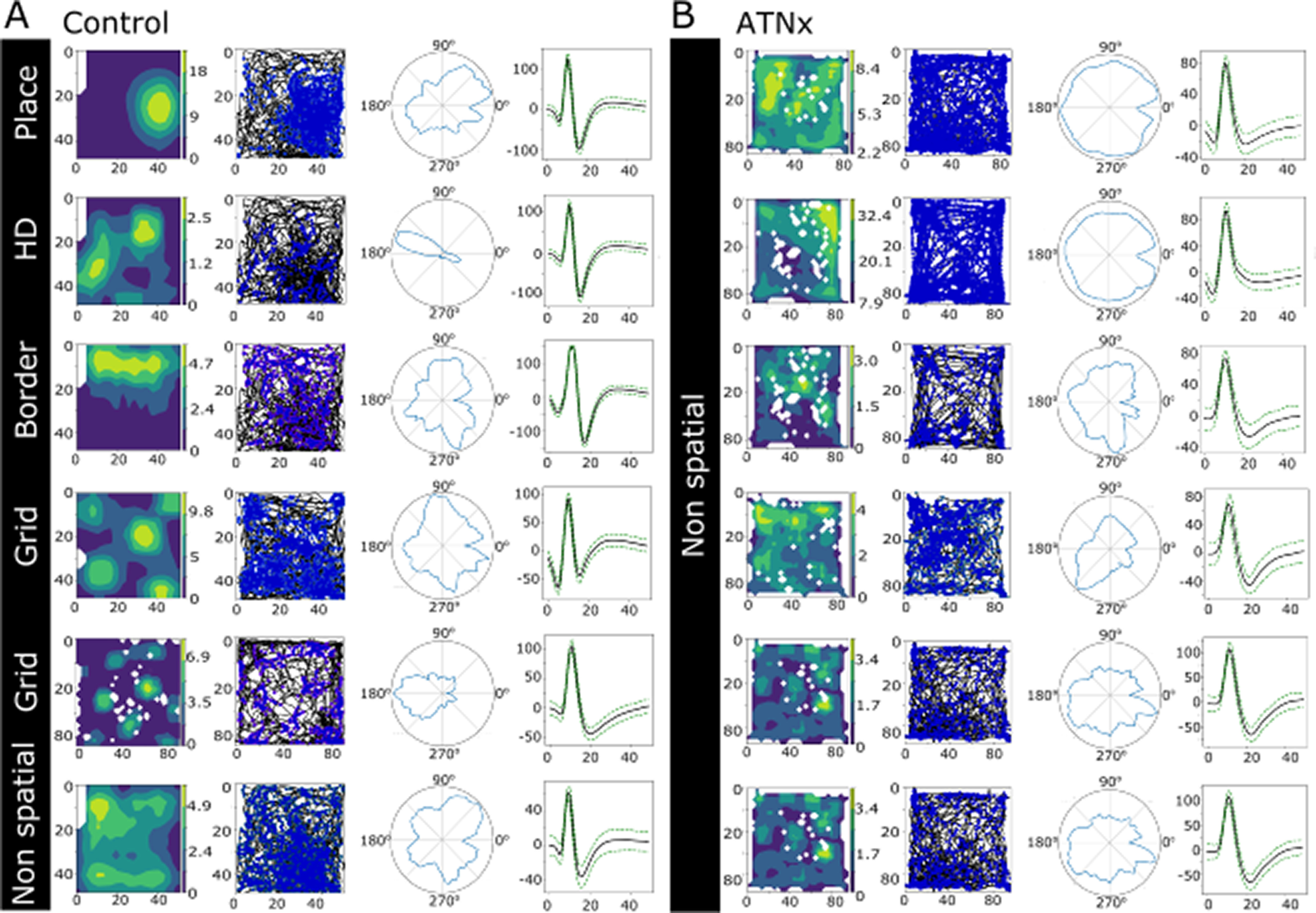
Representative single units recorded in the dorsal subiculum in control (Control; ***A***) and ATN lesion (ATNx; ***B***) animals. For individual units, the figures illustrate the following: heatmap of spike location adjusted for time spent in each location; path in arena (black) with spike location (blue); head direction; mean spike waveform. For the Control group, different classes of spatial cells are displayed, along with a nonspatial cell. No spatial cells were recorded in the ATNx cases. HD, Head direction.

Spatial and nonspatial single units were recorded in the dorsal subiculum of all animals. Of 82 single units recorded in the Control rats, 47 (57%) were considered spatial units (Fig. 3*A*). These units consisted of place (*n* = 11; 23%), head-direction (*n* = 20; 43%), border (*n* = 5; 11%), and grid (*n* = 11; 23%) cells. A further 35 (43%) did not show obvious spatial properties (e.g., no clear place field or preferred head direction). Strikingly, no spatial units were recorded in the ATNx animals, although nonspatial units (21), that is, those showing no preferential firing in specific place or head orientation, were present (Fig. 3*B*). Electrode tracks are reconstructed in Figure 1*F–H*.

In order to assess whether this absence of spatial cells in ATNx animals was statistically significant, recordings were selected from Control and ATNx animals that were deemed to be independent, that is, performed before and after electrode position was altered (during the habituation period), or were performed several days apart, as electrodes had likely shifted naturally. Recordings were considered to contain spatial cells or nonspatial cells, regardless of the number of cells recorded or whether the same cell had been recorded previously. The presence of spatial cells and nonspatial cells in the sampled recordings was accumulated in a contingency table (Table 2). The null hypothesis is that the true difference in proportion between the sample estimates is equal to 0. In other words, the percentage of recordings with cells of a certain type is independent of the condition being control or lesion. The results from running a two-sided Barnard's unconditional exact test (Barnard, 1947) for a binomial model on a 2 × 2 contingency table consisting of the observed frequencies from the Control and ATNx lesion recordings are as follows: for the spatial cells (Control 15 of 53 vs ATNx 0 of 47, Wald statistic 4.574, difference in proportion 0.28, *p* < 0.001), indicating the data strongly support rejecting the null hypothesis; for the nonspatial cells (Control 16 of 53 vs ATNx 13 of 47, Wald statistic 0.28, difference in proportion 0.0259, *p* = 0.81), indicating that the null hypothesis should not be rejected.

A Bayesian analysis was then conducted on the contingency table data (MacKay, 2003). We find that, at ∼99.99% chance, it is more likely to record a spatial cell in a Control recording, compared with an ATNx lesion recording, P(PC > PL) = 0.9999. Similarly, we find that, at ∼93.2% chance, it is 5 times more likely to record a spatial cell in a Control recording compared with an ATNx lesion recording, P(PC > 5 *PL) = 0.9328. Overall, we find spatial signaling is lost in the subiculum after ATN lesions.

### ATN lesions (NMDA) leave non-spatial subiculum firing properties largely unchanged

#### Spike properties

The mean spike width of all recorded subicular cells pooled into a single group was greater in ATNx animals than Control (Control 155.26 ± 50.43 µs, ATNx 213.88 ± 53.87 µs; W = 310, *p* < 0.001, Mann–Whitney *U* test). Other spike properties (Table 3, top section) showed no differences between groups, including number of spikes (Control 2694 ± 3250, ATNx 4718 ± 6326; *t*_(22.66)_ = −1.39, *p* = 0.179), spike frequency (Control 2.93 ± 4.31 spikes/s, ATNx 5.42 ± 7.07 spikes/s; w = 809, *p* = 0.673, Mann–Whitney *U* test), amplitude (Control 107 ± 31 µV, ATNx 104 ± 19 µV; *t*_(49.3)_ = 0.47, *p* = 0.642), and height (Control 147 ± 54 µV, ATNx 161 ± 39 µV; *t*_(40.74)_ = −1.34, *p* = 0.187). We tested the ISI medians for normality using the Shapiro–Wilk test, which indicated these data are not normally distributed (Control W = 0.530, *p* < 0.001 ATNx W = 0.691, *p* < 0.001). We therefore applied a nonparametric Mann Whitney *U* test to compare the ISI medians in the Control and ATNx groups. The means of the median ISIs in the Control and ATNx groups were 153 and 547 ms, respectively (Mann–Whitney W = 386, *p* = 0.010, two-tailed).

**Table 3.** Summary of spike and burst properties of subicular units

	Control		ATNx (lesion)	
	Mean	± SD	Mean	± SD
Number of spikes (per 20 min)	2694.93	3249.93	4718.00	6326.09
Spike rate (spikes/s)	2.93	4.31	5.42	7.07
Spike amplitude (µV)	106.91	31.15	104.33	19.20
Spike height (µV)	146.91	53.96	161.18	39.32
Spike width (µs)***	155.26	50.43	213.88	53.87
ISI (ms)	1008.59	1381.03	2253.24	3453.02
Total bursts (per 20 min)	238.90	435.98	116.6	157.78
Total bursting spikes (per 20 min)	526.59	981.86	242.90	331.59
Mean bursting ISI (ms)	4.08	0.32	3.88	0.28
Spikes per burst*	2.13	0.11	2.05	0.05
Mean burst duration (ms)*	5.61	0.64	5.07	0.45
IBI (ms)*	11144.49	11073.79	31830.77	39508.75
Mean duty cycle^*a*^ (burst duration/IBI)	0.05	0.05	0.02	0.02
Propensity to burst (bursting spikes/total spikes)***	0.12	0.09	0.06	0.03

^*a*^Duty cycle describes the portion of the IBI during which a burst fires.

*p < 0.05;

***p < 0.001; Welch's two-sample *t* test.

**Figure 4.**
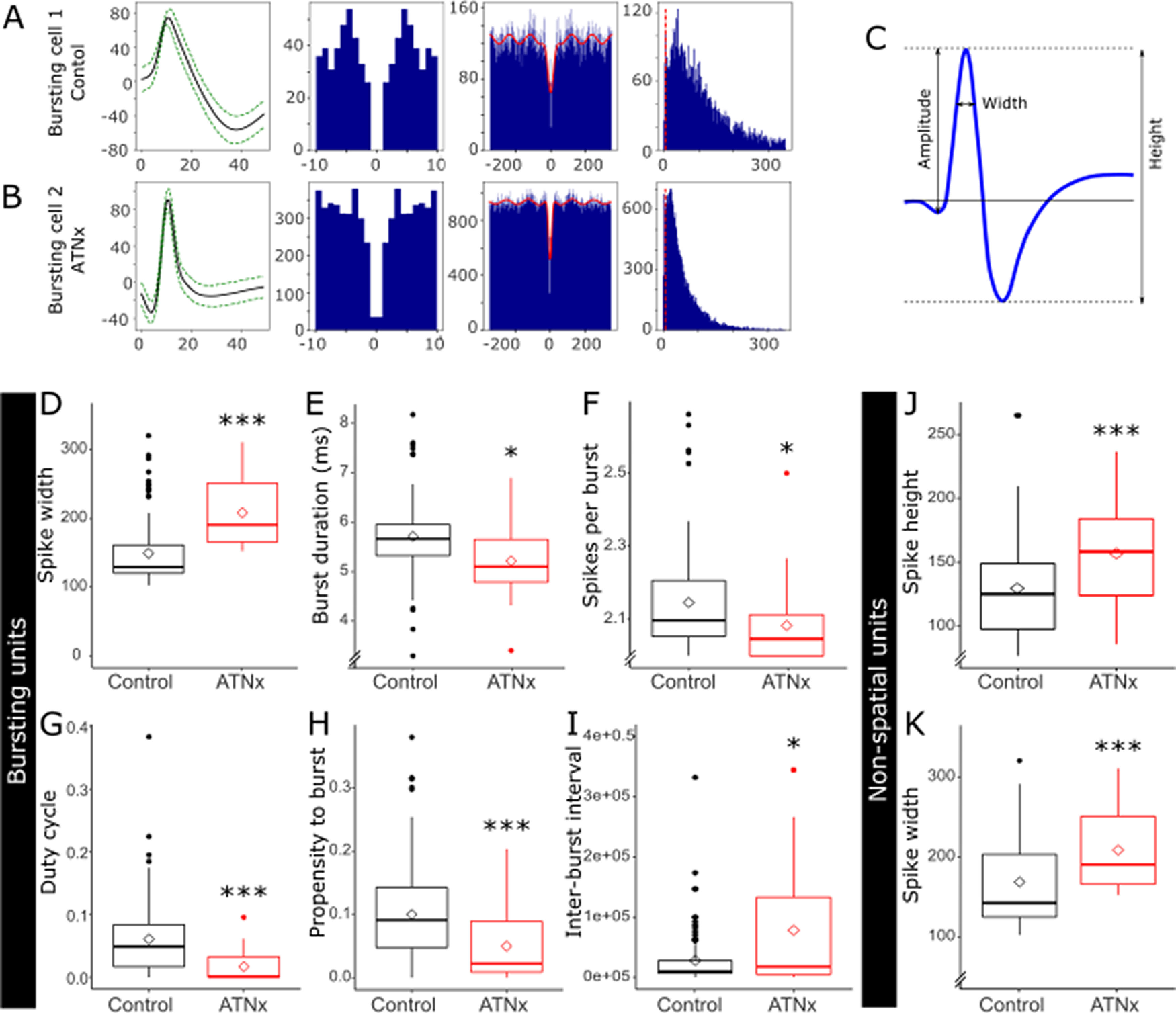
Properties of bursting and nonspatial subicular cells. ***A***, ***B***, Waveforms and autocorrelation histograms were used for cell classification. ***C***, Diagram of waveform properties. Bursting cells in Control showed a higher burst duration (***E***), had more spikes per burst (***F***), and had a higher propensity to burst (***H***). Bursting cells in ATNx had a greater spike width (***D***) and higher IBI (***I***), than nonbursting cells. ***J***, ***K***, Nonspatial cells in ATN had higher spike width and spike height than nonspatial cells in Control animals. ***D-K*** (boxplots), Filled circles represent outliers. Unfilled diamonds represent the mean. *p < 0.05; ***p < 0.001; Welch's two-sample *t* test or Mann–Whitney *U* test.

**Figure 5.**
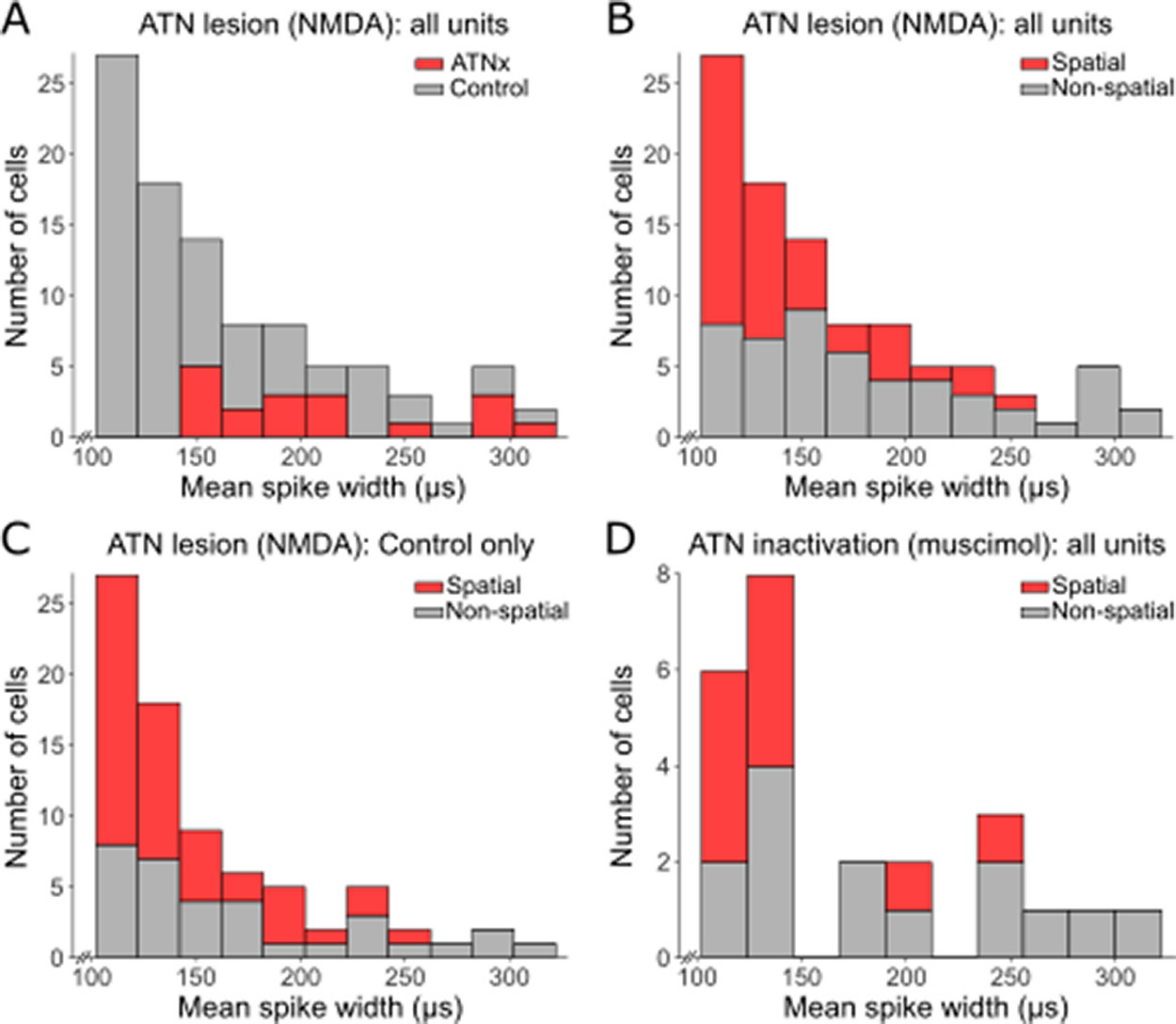
***A***, When Control rats are compared with rats with permanent NMDA lesions of the ATN (ATNx), more short-duration waveforms were recorded in Controls, but the samples obtained from the two groups of rats are not distinct. ***B***, ***C***, In the permanent lesion study, narrow waveform cells were more often spatial, but the classification of wide and narrow waveform cells was mixed. ***B***, Combined data for the Control and ATNx rats. ***C***, Data for only Control rats. ***D***, Similarly, in rats where the ATN was inactivated with muscimol, narrow waveform cells were more often spatial, but the groups were mixed.

When nonspatial cells in Control animals were compared with putative “nonspatial” cells in ATNx animals, spike width (Control 173 ± 60 µs, ATNx 213 ± 54 µs; *t*_(45.60)_ = −2.56, *p* = 0.014, Fig. 4*K*), and spike height (Control 133 ± 40 µV, ATNx 161 ± 39 µV; *t*_(42.11)_ = −2.57, *p* = 0.014, Fig. 4*J*) were both smaller in Control animals. Spike width was assessed further using histograms to determine whether the difference observed between Control and ATNx reflected different neuron populations (Fig. 5). More short-duration waveforms were recorded in Control animals than ATNx, but the samples obtained from the two groups were not distinct (Fig. 5*A*). Cells with narrow spike widths were more often spatial, but the classification of narrow and wide waveform cells was mixed in both Control and ATNx combined (Fig. 5*B*) and Control only (Fig. 5*C*). The mean width for controls of nonspatial cells was 170.6 ± 61.3 µs, while for spatial cells the mean was 142.5 ± 37.5 µs, a significant difference (Mann–Whitney *U* test, W = 962, *p* = 0.026).

#### Spike properties of bursting cells

As no spatial units were recorded in ATNx animals, it was unclear whether spatial cells were present but inhibited (and therefore not recorded) or whether the units that were recorded were latent spatial units that were now not responding to “spatial” inputs. Because of the difficulties in comparing nonspatial units in the Control to unknown spatial or nonspatial units in ATNx animals, subicular cells were classified according to their spike properties into bursting, fast spiking, and theta-entrained cells (Anderson and O'Mara, 2003). While the percentage of subiculum bursting cells in the Control (53% of 82 units) and ATNx (57% of 21) animals was essentially equivalent, other properties differed (Table 3, bottom section; Fig. 4). Cells in Control animals showed a greater propensity to burst than those in lesion animals (Control 0.12 ± 0.09, ATNx 0.06 ± 0.03; *t*_(35.46)_ = 3.69, *p* < 0.001; Table 3). Bursting cells in Control animals showed more spikes per burst (Control 2.13 ± 0.11, ATNx 2.05 ± 0.05; w = 1005, *p* = 0.004, Mann–Whitney *U* test) and greater burst duration (Control 5.61 ± 0.64 ms, ATNx 5.07 ± 0.45 ms; *t*_(18.21)_ = 3.04, *p* = 0.007), and conversely lesion animals showed larger IBIs than controls (Control 11,144 ± 11,074 ms, ATNx 31,831 ± 39,509 ms; *t*_(9.32)_ = −1.56, *p* = 0.038; Table 3).

### Muscimol infusion reversibly reduces spatial alternation memory performance to chance

**Figure 6.**
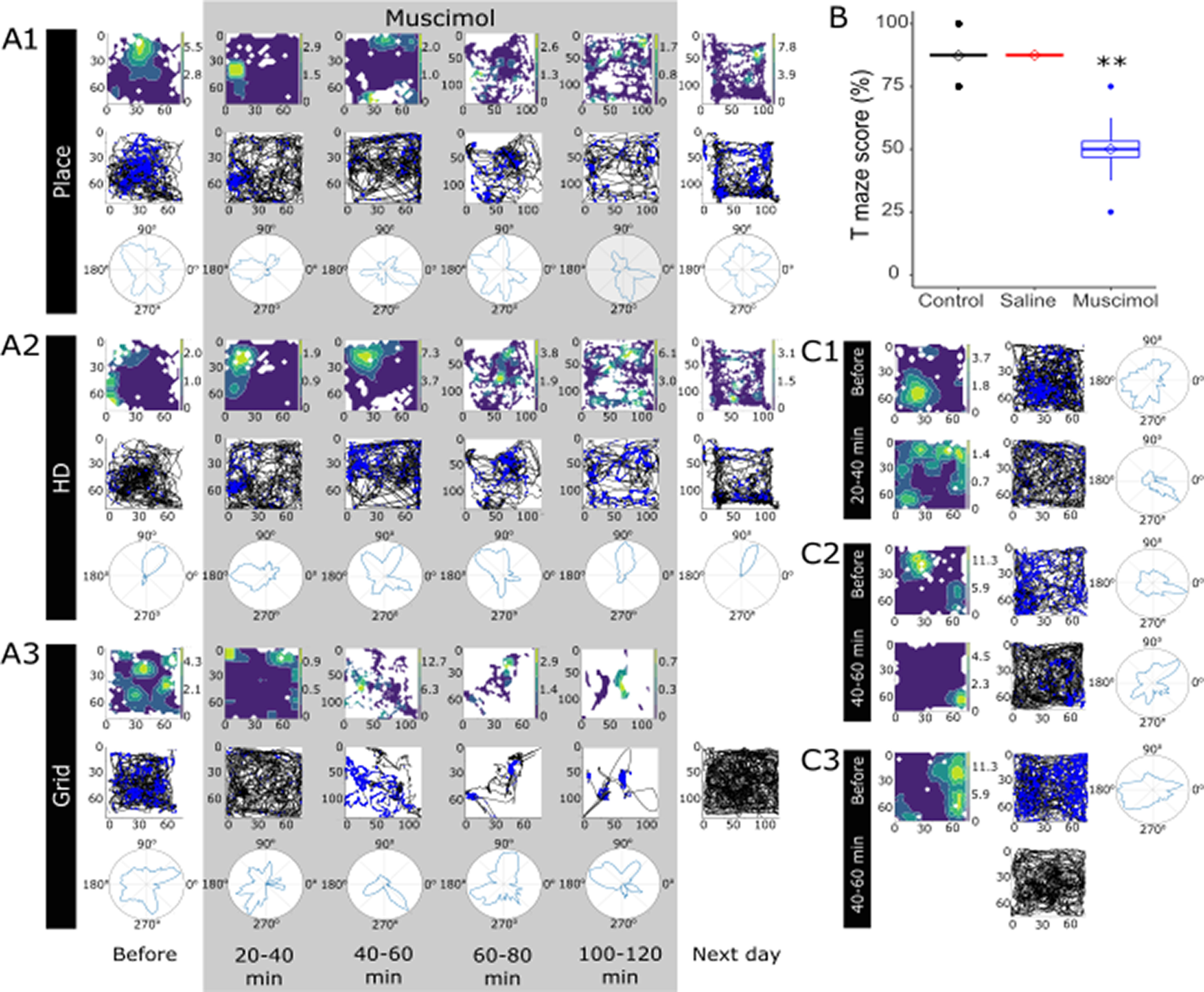
***A1-A3***, Examples of spatial units before, during, and after muscimol infusion. Spatial properties of single units decreased when ATN was inactivated. ***A3***, Relative inactivity after 100-120 min, and the cell was not recorded the next day. ***B***, Spatial alternation dropped to chance levels when the ATN were temporarily inactivated with muscimol, compared with the same animals before infusion. When saline was infused in place of muscimol, no deficit was present. ***C1-C3***, Further examples of spatial units before and after ATN inactivation. ***C1***, Disruption of place field shortly after muscimol infusion with some head directionality remaining, which was later disrupted. ***C3***, No firing was detected after muscimol infusion. **p < 0.01 (ANOVA with Tukey *post hoc*).

When the ATN was temporarily inactivated with muscimol, spatial alternation percentage dropped to chance levels (before muscimol infusion 87.27 ± 7.41; after muscimol infusion 50.00 ± 13.97; Fig. 6*B*). Two animals also received bilateral saline infusions as a control, showing no deficit in spatial alternation (87.5 ± 0%). The muscimol inactivation caused a significant deficit in spatial alternation compared with both before muscimol and saline infusion (ANOVA, *F*_(3, 62)_ = 49.69, *p* < 0.001, Tukey *post hoc p* < 0.001).

### Muscimol infusion reversibly abrogates subiculum spatial firing

Before the muscimol infusion sessions, electrophysiological recordings were conducted on cannulated rats to allow for electrode adjustment and habituation to recording equipment. Single units were recorded during these habituation periods; however, only units recorded during the inactivation experiments were considered. Thirty-five cells were recorded during muscimol experiments. Of these, 29 were recorded at baseline (i.e., immediately before muscimol infusion) and so included in the study (Fig. 6*A1-A3*,*C1-C3*). Of the 29 recorded at baseline, only one cell was recorded before infusion and not afterward.

### Muscimol infusion leaves nonspatial subiculum firing properties largely unchanged

#### Spike properties

For analysis, unless otherwise stated, Welch's two-sample *t* tests were performed to consider cell parameters with respect to baseline data, with Bonferroni *post hoc* correction to account for multiple comparisons of time points to baseline. When all cells were grouped together, there was no significant change in mean spiking frequency following ATN inactivation (baseline 5.07 ± 9.10 spikes/s, 40-60 min after infusion 2.90 ± 3.84 spikes/s; *t*_(45.21)_ = 0.21, *p* = 0.230), or the number of spikes per recording (baseline 5306 ± 10,344, 40-60 min after infusion 3483 ± 4607; *t*_(46.48)_ = 0.89, *p* = 0.378). Mean spike width (baseline 180.32 ± 55.20 µs, 40-60 min after infusion 185.81 ± 50.71 µs; *t*_(55.53)_ = 0.39, *p* = 0.698) and amplitude (baseline 113.01 ± 41.43 µV, 40-60 min after infusion 115.22 ± 45.48 µV; *t*_(51.13)_ = 0.19, *p* = 0.851) showed no change following ATN inactivation when all cells were grouped. A histogram of spike widths showed that, while cells with narrow waveforms were more often spatial, the spatial and nonspatial groups remained mixed (Fig. 5*D*). Finally, when all units were grouped, temporary ATN inactivation led to an apparent doubling of the ISI (baseline 664.56 ± 736.57 ms, 40-60 min after infusion 1443.29 ± 1825.34 ms), although this was not significant (w = 351, *p* = 0.239, Mann–Whitney *U* test).

Following muscimol infusion, spatial properties of subiculum cells declined, despite no decrease in firing frequency. For designated place cells before infusion, the place field became disrupted after ATN inactivation (Fig. 6*A1*). Head directionality also became disrupted without ATN input (Fig. 6*A2*), and grid cells did not fire in a grid-like pattern (Fig. 6*A3*). In most cases, these spatial properties were recovered by the following day, although in some cases the cells could no longer be recorded (Fig. 6*C3*). Interestingly, the subiculum grid cells appeared to lose their place field initially but retained some head directionality, before this too was disrupted (Fig. 6*A3*,*C1*,*C2*). We performed an additional control measure by spatially downsampling the firing maps before and after muscimol injection to match occupancy in each spatial bin.

#### Spike properties of subiculum bursting cells

**Figure 7.**
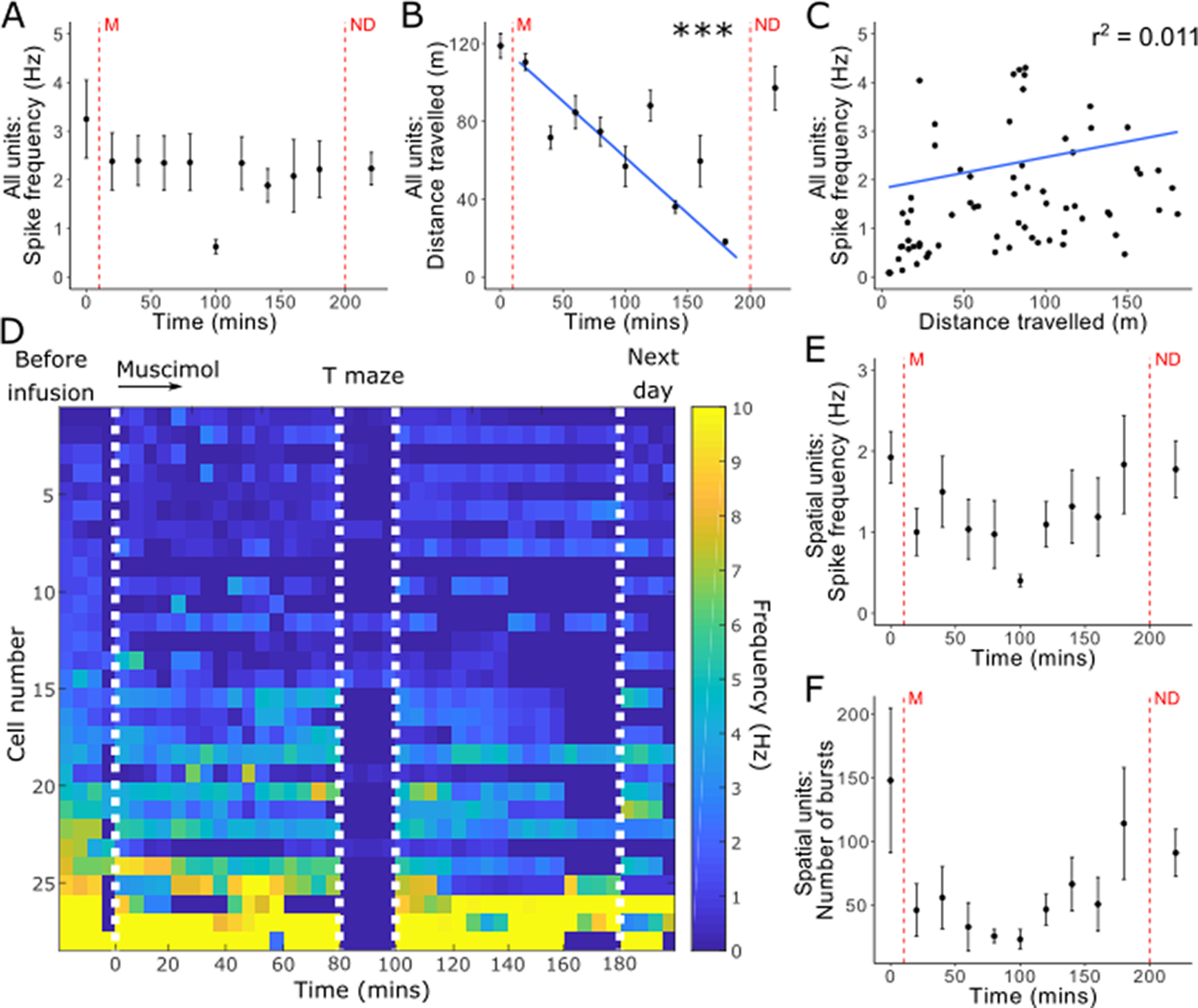
Spike properties following temporary inactivation of ATN with muscimol (M). ***A***, Inactivation of ATN caused no significant decrease in single-unit firing frequency. ***B***, Animals showed decreasing levels of activity throughout the experiment, although there was no significant correlation between distance traveled and spike frequency (***C***). ***D***, Firing frequency of each cell recorded at baseline (left), in 5 min bins throughout the experiment. First white line indicates ATN inactivation with muscimol, after 15-20 min of baseline recording before infusion. In most cases, electrophysiological recording was paused for T-maze testing between 80 and 100 min (second and third white lines), then continued. Recordings in which the animal was largely inactive or asleep were excluded. Final white line indicates data from the day after infusion. ***E***, There were no significant changes in spike firing in spatial units as a result of ATN inactivation and burst properties remained consistent, including the number of bursts (***F***). ***A***, ***B***, ***E***, ***F***, First red vertical line (M) indicates the infusion of muscimol. Second red vertical line indicates recordings taken the next day (ND). Each bin represents 20 min of recording. Data are compared with baseline, immediately before inactivation, with error bars indicating SEM. Theta-entrained cells are removed from ***A***, ***B***, ***C*** because of high firing frequency compared with other cell classes. ***p < 0.001 (Welch's two-sample *t* test with Bonferroni correction).

Of the 29 units included in the study, at baseline, 20 were classified as bursting, 6 fast spiking, and 2 theta-modulated (before muscimol infusion). For spike property analysis, theta-modulated units were excluded because of their high firing frequency and insufficient numbers to perform further statistical analysis. When fast spiking and bursting units were combined, there was no difference in firing frequency compared with baseline, following ATN inactivation with muscimol (5.07 ± 9.10 spikes/s, 0-20 min after infusion 3.20 ± 4.75 spikes/s; *t*_(52.31)_ = 0.87, *p* = 0.385; Fig. 7*A*). All animals traveled less distance (i.e., showed less activity) as time increased across the experiment (baseline, 115.97 ± 33.12 m; 60-80 min, 58.17 ± 37.41 m; Fig. 7*B*), although there was no correlation between firing frequency and distance traveled (*r*^2^ = 0.011; Fig. 7*C*). Figure 7*D* illustrates the firing frequency of each cell at baseline, then its frequency in 5 min intervals following muscimol infusion and again the following day, in those instances where the cell was recorded again.

Bursting units did not show any changes in burst properties following inactivation of the ATN, including number of bursts, spikes per burst, and burst duration (*p* > 0.05). Cells that had been designated as spatial units before infusion showed no changes in burst properties or firing frequency, indicating that these spatial cells continue to fire following ATN inactivation but no longer displayed spatial properties.

### Anterior thalamic lesions (NMDA) do not affect CA1 place cells but reduce spatial alternation performance to chance

To explore the effects of anterior thalamic lesions on spatial processing in the dorsal hippocampus, a further 3 rats were implanted with a microdrive apparatus with 8 recording tetrodes into the dorsal CA1 and received bilateral, permanent (NMDA) lesions of the ATN (ATNx_CA1). As in the previous experiment, ATNx_CA1 lesions were quantified by comparing anti-NeuN-reacted cell counts in the ATN with those from Control. In all ATNx_CA1 animals, the surgery consistently produced a marked cell loss throughout almost the entire ATN. An independent-sample *t* test showed significant difference in anti-NeuN cell counts between ATNx_CA1 (4166 ± 1003) and Control (16,209 ± 2507; *t*_(7)_ = 9.08, *p* < 0.001) groups.

#### Spatial alternation deficits

**Figure 8.**
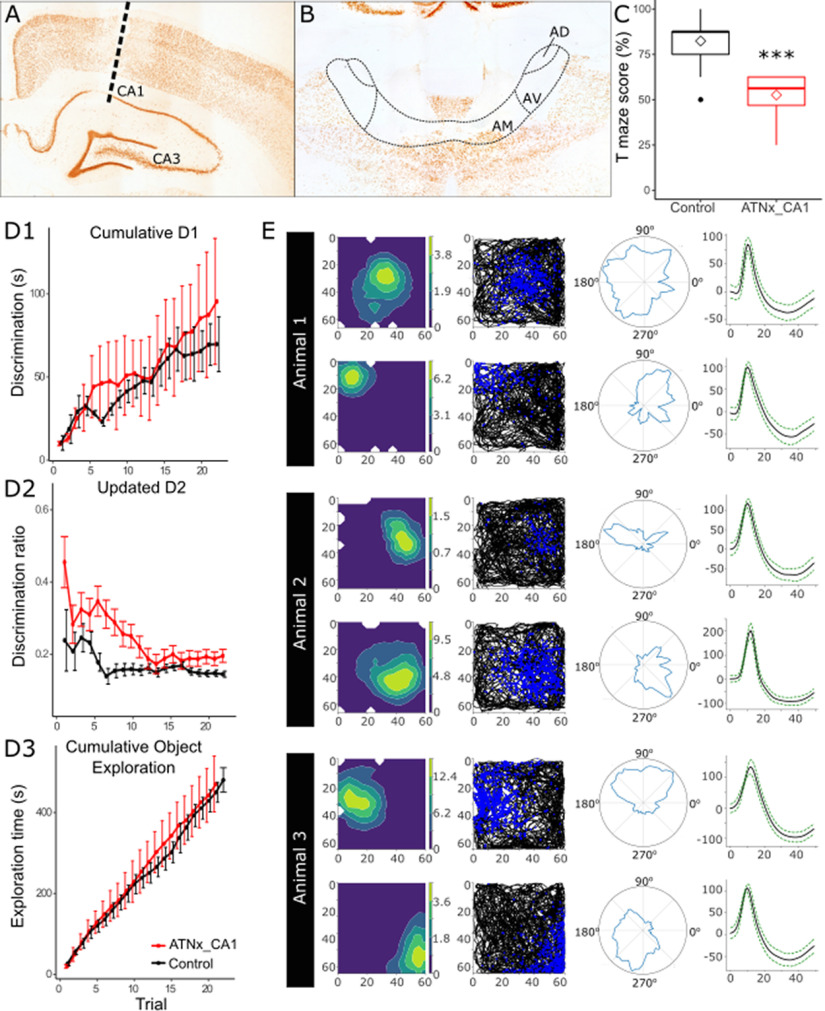
Representative electrode placement in CA1 (***A***) and ATN lesion (***B***), in NeuN-reacted sections. ***C***, Animals with ATN lesions and electrodes implanted in CA1 showed a significant deficit in spatial alternation task compared with Control animals (control data repeated from Experiment 1). ***D1-D3***, The same cohort of ATNx animals showed no deficit in object recognition on bow-tie maze. ***E***, Representative place cells recorded from CA1 in 3 ATNx animals. ***p < 0.001 (Welch's two-sample *t* test).

As expected, ATNx_CA1 rats showed a deficit in spatial alternation on the T-maze, equivalent to ATNx animals (Control 82.38 ± 11.18, ATNx_CA1 52.60 ± 11.40, *t*_(40.93)_ = −10.71, *p* < 0.001; Fig. 8*C*), confirming an impairment in spatial working memory. These animals also showed no deficit in object recognition and no difference in overall exploration time, compared with Control (*p* > 0.05; Fig. 8*D1–D3*).

### CA1 place cells are unaffected by ATN lesions

Daily recordings were conducted in CA1 of ATNx animals (ATNx_CA1) performing a pellet-chasing task in an arena. From these animals, 203 well-isolated units were recorded from dorsal CA1. Units were further classified into 107 spatial units using sparsity and coherence criteria (Schoenenberger et al., 2016) (Rat 1, *n* = 12; Rat 2, *n* = 67; Rat 3, *n* = 28). Putative interneurons were considered to have been recorded from dorsal CA1 if they were recorded on the same tetrode and in the same recording session as a spatial unit. Despite the absence of spatial signals in the subiculum, hippocampal (CA1) place cells appeared intact (Fig. 8*E*).

## Discussion

The origins of diencephalic amnesia remain something of a mystery, despite being described over a century ago (Arts et al., 2017), starkly contrasting with hippocampal amnesia, which has been investigated in breadth and depth since the 1950s (Scoville and Milner, 1957). Clinically, diencephalic amnesia presents as a dense anterograde amnesic syndrome, paralleling the amnesia associated with bilateral hippocampal damage (Arts et al., 2017); the ATN appear to be critical structures (Harding et al., 2000; Kril and Harper, 2012). Here, we based our rationale on how the ATN project to the hippocampal formation (specifically, the subiculum), but not the hippocampus proper (which includes area CA1). After ATN lesions, CA1 place cell activity appeared preserved, whereas the usual heterogeneous subicular spatial activity (place, head-direction, grid, and border cells) was striking by its absence. Moreover, after ATN lesions, performance fell to chance on a spatial memory task but remained intact on a nonhippocampal object recognition memory task (Albasser et al., 2012), indicating a specific, rather than generalized, deficit. The same disruptive effects (behavioral; cellular firing) proved reversible when using transient inactivation (muscimol) of the ATN. Thus, we conclude that a key contributor to diencephalic amnesia stems from the loss of anterior thalamic influence on the output regions of the hippocampal formation (Nelson et al., 2020) and, specifically, on the subiculum.

The behavioral effects of our ATN lesions corresponded to those of hippocampal lesions: severely impaired T-maze alternation, but spared object recognition memory in the bow-tie maze (Albasser et al., 2010). ATNx rats showed increased motor activity in square arenas, matching prior evidence of activity increases following ATN lesions in spatial settings (Warburton and Aggleton, 1999; Poirier and Aggleton, 2009; Dumont and Aggleton, 2013). Our extensive ATN lesions largely left CA1 place cells intact (there are some reports of microstructural changes and alterations in neuronal activity in CA1 following ATN disruption; Calton et al., 2003; Dillingham et al., 2019). Notably, the proportions of CA1 place cells in ATNx_CA1 animals is in line with our previous experiences of normal animals (Wang et al., 2012; Hok et al., 2012) and that of others (Thompson and Best, 1989; Meshulam et al., 2017; Duvelle et al., 2019; Broussard et al., 2020), contrasting with the more disruptive effects of medial entorhinal cortex lesions on CA1 place cell activity (Hales et al., 2014). As the majority of pyramidal cells in CA1 are place cells, and CA1 heavily innervates the subiculum, it could be assumed that subiculum spatial cells are principally driven by their CA1 inputs, especially as this projection shows activity-dependent plasticity (Commins et al., 1998a,b; Commins and O'Mara, 2000). The present study revealed, however, CA1 projections alone do not support subicular spatial firing or spatial alternation memory. Rather, ATN projections (presumably direct and indirect) are crucial for subicular spatial cellular discharge, as well as for spatial alternation memory.

The anteroventral nucleus possesses theta-modulated head-direction cells (Tsanov et al., 2011), and the anteromedial nucleus contains place cells and perimeter/boundary cells (Jankowski et al., 2014; Matulewicz et al., 2019). Consistent with previous reports (Sharp and Green, 1994; Brotons-Mas et al., 2010, 2017), we found spatial cells (including grid-like cells) in the dorsal subiculum of control animals. Although grid cells have been widely studied in the hippocampal formation since their discovery (Hafting et al., 2005), the presence of grid cells in the dorsal subiculum is still a relatively new finding (Brotons-Mas et al., 2017). Grid cells have been reported in the presubiculum and parasubiculum (Boccara et al., 2010), both parahippocampal areas receiving anterior thalamic inputs (van Groen and Wyss, 1990). Anterior thalamic lesions disrupt grid cell (and head-direction) activity in these same parahippocampal areas (Goodridge and Taube, 1997; Winter et al., 2015). The grid-like signal found in presubiculum and subiculum might have thalamic (Goodridge and Taube, 1997) and entorhinal (Hafting et al., 2005) components.

Stewart and Wong (1993) originally observed *in vitro* that subicular cells can be classified into bursting and nonbursting classes (confirmed by Sharp and Green, 1994; *in vivo*). A fuller *in vivo* analysis subsequently concluded subicular units could be classified into bursting, regular spiking, theta-modulated, and fast spiking units (Anderson and O'Mara, 2003). Sparsely bursting subicular cells potentially carry more spatial information (Simonnet and Brecht, 2019). Here, although the proportion of bursting cells in control and ATNx groups remained equivalent, some properties differed: controls showed a greater burst propensity, more spikes per burst, and greater burst duration. Conversely, lesioned animals showed somewhat larger IBIs than controls. This alteration in bursting may affect the fidelity of subicular-retrosplenial transmission, as bursting cells in dorsal subiculum with direct connections to granular RSC directly impact sharp-wave ripples in RSC (Nitzan et al., 2020).

Given the routes of ATN fibers reaching the medial temporal lobe, it is worth considering whether we have recorded from fibers of passage. *In vitro*, “direct recording of single AP (action potential) transmission is challenging” because of the small diameters of axons and recording instability (Radivojevic et al., 2017). However, Robbins et al. (2013) report, in performing hippocampal and alvear recordings, the presence of short-duration triphasic waveforms having a peak-trough length of <179 μs, recorded mostly on only one wire of a tetrode. While we recorded for long durations in freely behaving, implanted animals, our units have a half-width of ∼0.8–1.2 ms, within the refractory period for action potentials. On occasion, we might possibly have recorded from such fibers (e.g., Fig. 3*A*, top grid cell; but compare Brotons-Mas et al., 2017; Figs. 3*B*, 4*C*). The possibility of recordings of fibers of passage in subiculum needs further investigation. A different concern relates to the disruption of thalamic fibers of passage. Here, muscimol helped to confirm the NMDA lesions targeted ATN neurons, and not fibers of passage (Winter et al., 2015).

The subiculum is the primary hippocampal output of area CA1 (Amaral et al., 1991; O'Mara et al., 2001, 2009; Cembrowski et al., 2018), an area with substantial numbers of place cells, suggesting CA1-subiculum inputs are principally spatial. Our data cast new light on this relationship, as CA1 inputs are not sufficient to ensure subicular spatial firing. One possibility is that the ATN normally exert a direct modulatory, including oscillatory (Vertes et al., 2001), influence on the subiculum that, when removed, leads to changes in gain control, disrupting information processing. Given the range of spatial cells in the ATN (Taube, 1995; Jankowski et al., 2015; Matulewicz et al., 2019), such an input might, for example, help hippocampal and parahippocampal regions coregister their various spatial signals, including those from CA1. This might explain why ATN lesions increased dorsal subiculum spike width and reduced bursting properties, but left other features seemingly intact (e.g., overall spike frequency and amplitude). The latter findings help explain why immediate-early gene analyses have found that ATN lesions can cause hypoactivity in parahippocampal fields, but little apparent impact on the dorsal subiculum (Dillingham et al., 2019).

The present findings invoke a wider network account of ATN loss that includes direct efferent actions on the subiculum, alongside indirect actions via parahippocampal and retrosplenial targets. There remains, however, the intriguing issue of why other areas (e.g., CA1, entorhinal cortex, and their outputs) are insufficiently independent of the ATN to preserve subicular spatial firing and ensure effective spatial alternation memory. One clue is the subiculum sends very dense direct and indirect projections to the ATN; that is, it is a key part of a complex, reciprocal, set of pathways (Bubb et al., 2017). Furthermore, direct anterior thalamic-hippocampal interactions are needed for spatial alternation, regardless of whether they are ATN projections to the dorsal hippocampus (including subiculum, as examined in the present study), or dorsal subicular projections to the ATN (Nelson et al., 2020). Understanding this reciprocal relationship will prove integral to understanding why ATN damage is such a critical component of diencephalic amnesia (Aggleton and Brown, 1999).

The present results demonstrate the critical roles for the ATN in navigation and spatial learning, and spotlight the pivotal role of the subiculum, given the severity of the spatial memory/alternation deficit and the concurrent sparing of CA1 place cells. As the subiculum is a principal source of hippocampal projections beyond the temporal lobe, these findings reveal how anterior thalamic damage might indirectly affect sites, such as the mammillary bodies, ventral striatum, and medial and orbital prefrontal cortices (Aggleton et al., 2015; Ferguson et al., 2019; Haugland et al., 2019). These data also indicate that signaling from within the hippocampus (e.g., CA1) is not sufficient to support the varied spatial signals found in the subiculum. Subicular spatial signals might arise from converging inputs from CA1, the ATN, and parahippocampal areas, including entorhinal cortex. Further work should determine the computations performed by the subiculum on the inputs it receives from ATN to support spatial alternation performance, and to discover how the substantial input from hippocampal area CA1 is gated by ATN inputs reaching the subiculum. Similar considerations suggest a complex origin for diencephalic amnesia, which partly arises from the direct and indirect loss of ATN inputs to output regions of the hippocampal formation.
